# Velocity-dependent friction enhances tribomechanical differences between monolayer and multilayer graphene

**DOI:** 10.1038/s41598-019-51103-1

**Published:** 2019-10-10

**Authors:** F. Ptak, C. M. Almeida, R. Prioli

**Affiliations:** 1 Departamento de Física, Pontifícia Universidade Católica do Rio de Janeiro, Marques de São Vicente 225, Rio de Janeiro, 22453-900 Brazil; 2 Divisão de Metrologia de Materiais, Instituto Nacional de Metrologia, Qualidade e Tecnologia (INMETRO), Av. Nossa Senhora das Graças 50, Xerém, Duque de Caxias, Rio de Janeiro, 25250-020 Brazil

**Keywords:** Mechanical and structural properties and devices, Surfaces, interfaces and thin films

## Abstract

The influence of sliding speed in the nanoscale friction forces between a silicon tip and monolayer and multilayer graphene were investigated with the use of an atomic force microscope. We found that the friction forces increase linearly with the logarithm of the sliding speed in a highly layer-dependent way. The increase in friction forces with velocity is amplified at the monolayer. The amplification of the friction forces with velocity results from the introduction of additional corrugation in the interaction potential driven by the tip movement. This effect can be interpreted as a manifestation of local thermally induced surface corrugations in nanoscale influencing the hopping dynamics of the atoms at the contact. These experimental observations were explained by modeling the friction forces with the thermally activated Prandtl-Tomlinson model. The model allowed determination of the interaction potential between tip and graphene, critical forces, and attempt frequencies of slip events. The latter was observed to be dominated by the effective contact stiffness and independent of the number of layers.

## Introduction

Graphene, a layered material composed of carbon atoms structured on a hexagonal lattice, has attracted much attention in the scientific community^1^, being a candidate for the fabrication of electronic devices^2^, gas sensors^3^ as well as micro- and nano-electromechanical systems (MEMS and NEMS, respectively)^4,5^. Although the tribological properties of graphene have not yet been fully understood, the usage of graphene as both solid lubricant and coating has been proposed^6–10^. Studying friction in graphene becomes even more relevant as tribological properties of nanoscale materials might differ considerably from their bulk counterparts^11^, presenting novel and unexpected features.

Many efforts have been reported on this issue, from both experimental^6,12–21^ and theoretical^15,22–26^ approaches. Such studies show that friction in graphene is influenced by parameters such as the number of graphene layers^12,15^, interaction with the substrate^14,15,19^, surface roughness^25,27,28^ and crystallographic orientation^17,21,29,30^. The number of layers has an important role in the friction mechanism of graphene, as friction was observed to decrease with the increasing number of layers. This was first attributed to the electron-phonon coupling^12^ and later explained by the out-of-plane deformations in the graphene sheets^13–15^. The interaction with substrate is of relevance as graphene deposited on atomically thin materials has shown much lower friction than graphene deposited on silicon substrates^15^. Such interaction influences the surface roughness, diminishing it and lowering the friction forces^28^. The crystallographic orientation of the graphene sample is also important, as the energy dissipation along the *armchair* direction can be higher than along the *zigzag* direction for a single-layer graphene^21^. The different crystallographic orientations have also been associated with friction domains that may arise during scanning^29^.

The sliding speed between contacts is of importance in friction mechanisms and has been broadly studied^31–36^. Although there is a significant influence of the sliding speed in friction for bulk materials, only a few studies measuring its influence in friction on atomically thin layered materials have been reported^24,26,37^. Computer simulations in graphene reveal either a non-linear dependence of friction with the logarithm of the sliding speed^24^ or no influence of speed in friction^26^. Experiments with hexagonal boron nitride shows an exponential dependence in friction with the sliding speed in the low velocity range^37^.

In atomic scale, dry friction is often described by the thermally activated Prandtl-Tomlinson model (PT model)^38,39^. Therein, the tip-sample interaction potential *V(x,t)* is considered as a combination of a periodic potential due to the crystalline nature of the sample surface and the elastic potential energy stored by the cantilever, given by:

1 $$V(x,t)=-\frac{{V}_{0}}{2}\,\cos (\frac{2\pi x}{a})+\frac{{k}_{eff}}{2}{(vt-x)}^{2}.$$

In Eq. (1), *V*_0_ is the amplitude of the periodic potential, *k*_*eff*_ the effective contact stiffness, *v* is the scanning velocity and *a* is the sample’s lattice parameter. The tip is modeled as a mass-spring system and moves in a stick-slip mode. As the tip scans the surface, it will eventually be in a minimum potential well while friction forces extend the spring, as the tip support is continuously scanned, thus increasing its energy. The spring is extended until the cantilever tip has enough energy to surpass a potential barrier, slipping into the next potential well. The barrier *ΔV* is defined as *ΔV* = *V*(*x*_*max*_, *t*) − *V*(*x*_*min*_, *t*), where *x*_*max*_ and *x*_*min*_ give the maxima and minima of the potential *V(x*, *t)* at a time *t*, and can be parametrized in terms of the friction force *f*_*L*_ while *F*_*c*_ is a critical force at which the barrier vanishes^40^, given by:

2 $$\Delta V={V}_{0}{(1-\frac{{f}_{L}}{{F}_{c}})}^{3/2}$$

In this model, at a thermal energy *kT* the slip process is probabilistic^31–33^, associated with a velocity *v*_0_ and an attempt frequency *f*_0_ related by:

3 $${v}_{0}=\frac{2kT{F}_{c}}{3{V}_{0}{k}_{eff}}{f}_{0}$$

The slip process is then governed by the relation between friction force and scanning velocity^33,41^:

4 $$\mathrm{ln}\,\frac{v}{{v}_{0}}=-\frac{\Delta V}{kT}-\frac{1}{2}\,\mathrm{ln}(1-\frac{{f}_{L}}{{F}_{c}})$$

In this work, we investigate the influence of scanning speed on friction forces in an atomically-thin layer material. Our results show a layer dependency in friction, as the increase of friction with velocity is higher for a monolayer than for multilayer graphene. The effective contact stiffness between the cantilever tip and graphene was observed to be independent on the number of layers. We were able to interpret our results with the PT model, extracting relevant parameters for the cantilever-graphene system.

## Results

### Velocity dependence of friction forces in graphene

**Figure 1:**
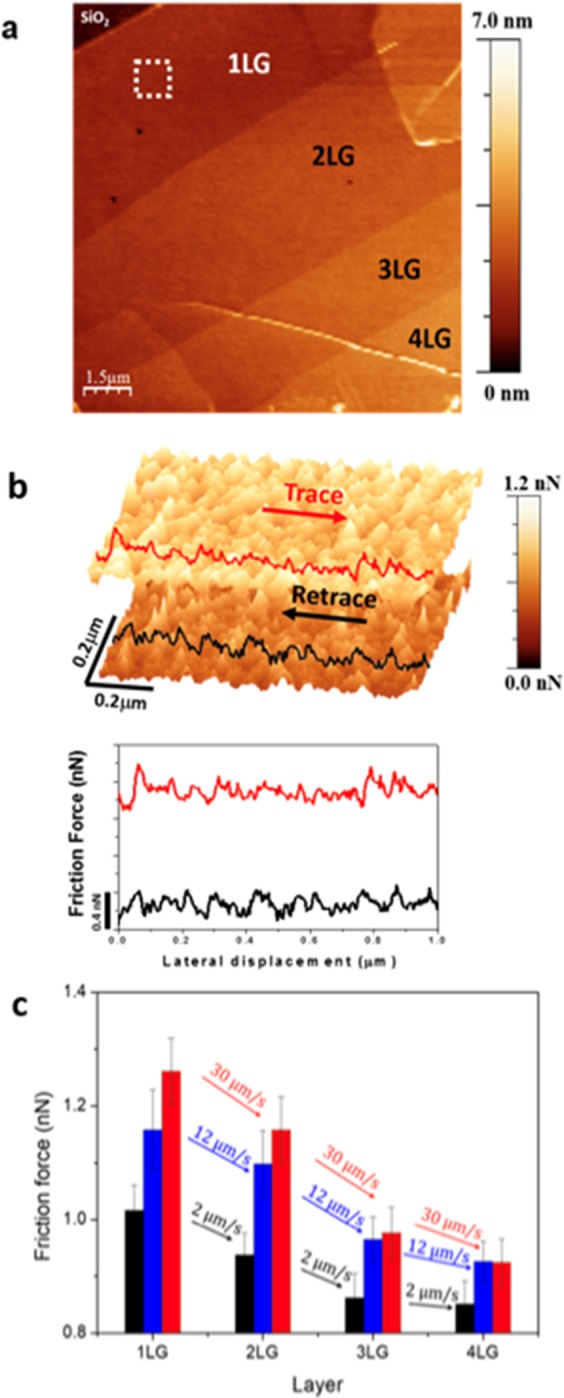
(**a**) Topography image of a graphene flake with one to four layers; (**b**) 1 µm × 1 µm friction force forward and backward images and a friction-loop; (**c**) Measured friction force for different graphene layers at different scan velocities. Velocities are indicated by the arrows. Error bars are the standard deviation of measurements.

Friction in graphene is layer dependent. Figure 1(a) shows an AFM topography image of a graphene sample containing one to four layers. The height between substrate and first layer was measured as ∼0.8 nm while between the successive graphene layers were observed to be ∼0.5 nm as measured from the histogram of height distribution from topography images (details in supplementary material). On each graphene layer, a region of interest, represented by the dashed square in Fig. 1(a), was selected for further imaging and analysis of the friction force loops as in Fig. 1(b). Figure 1(c) shows the measured friction forces for the different graphene layers at three different scanning velocities, indicated by the arrows. There is a clear trend of decreasing friction with increasing number of layers and, as the scanning speed increases, friction is observed to increase. That increase in friction with speed is more pronounced at the monolayer than for the multilayer graphene.

**Figure 2:**
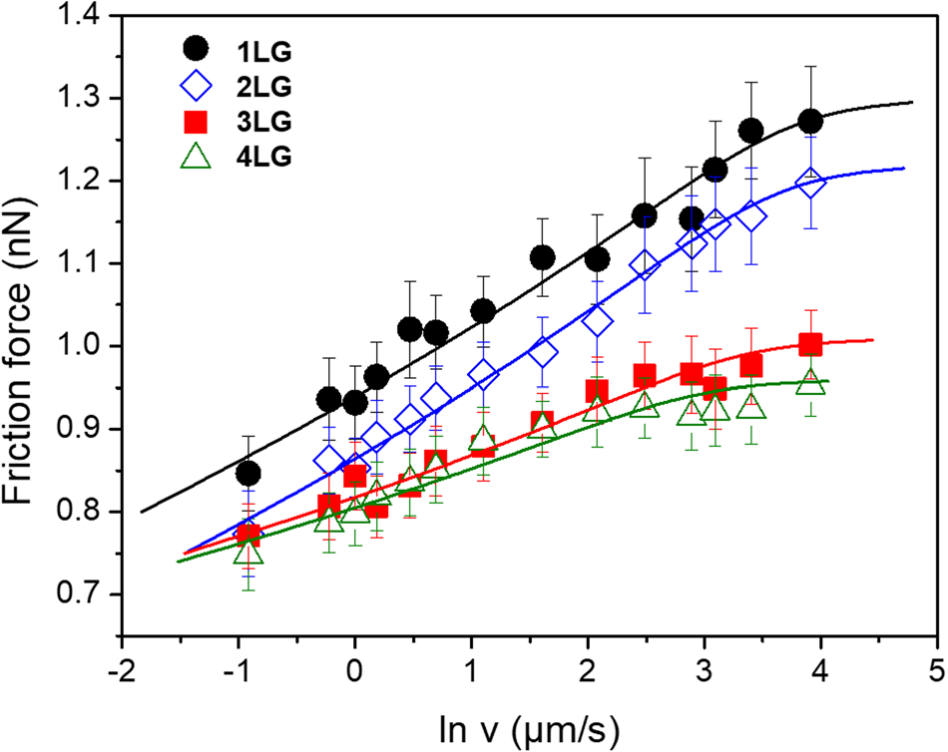
Measured friction force as a function of the logarithm of the scanning velocity for different graphene layers. Fitted curves are presented as solid lines. Error bars are the standard deviation of measurements.

Figure 2 shows the measured friction forces with respect to the natural log of the scanning velocity. Fitted curves with Eq. (4) are shown as solid lines in Fig. 2 and the parameters obtained are summarized in Table 1. The influence of velocity on friction at the monolayer and bilayer is significant, as indicated by the high slopes and velocities (∼30 µm/s) necessary to observe saturation, where friction forces becomes constant at a critical value. The curves for three- and four-layer graphene show lower slopes and velocities (∼10 µm/s) at saturation. Additionally, the energy dissipated by the tip during scanning was calculated. It is directly proportional to the mean friction force, thus following a similar trend with scanning velocity as friction. At top scanning speed (∼50 µm/s), we calculated energies up to ∼2.0 eV for 1LG and ∼1.5 eV for 4LG (supplemental material).

**Table 1:** Parameters obtained with the data fitting by Eq. (4).

Layer	*ΔV/kT*	*F*_*c*_ (nN)	*ln v*_0_ (µm/s)	*f*_0_ (MHz)
1LG	18 ± 3	1.33 ± 0.08	2.0 ± 1.0	1.8 ± 0.8
2LG	16 ± 2	1.22 ± 0.03	1.9 ± 0.4	1.6 ± 0.6
3LG	28 ± 6	1.01 ± 0.01	1.5 ± 0.5	2.1 ± 0.9
4LG	30 ± 10	0.96 ± 0.01	1.0 ± 0.6	1.6 ± 0.8

Errors are confidence interval of the fitting results. The attempt frequency *f*_0_ was calculated with Eq. (3). Errors in *f*_0_ are calculated as standard deviation in error propagation theory.

### Stochastic nature of friction forces in multilayer graphene

**Figure 3:**
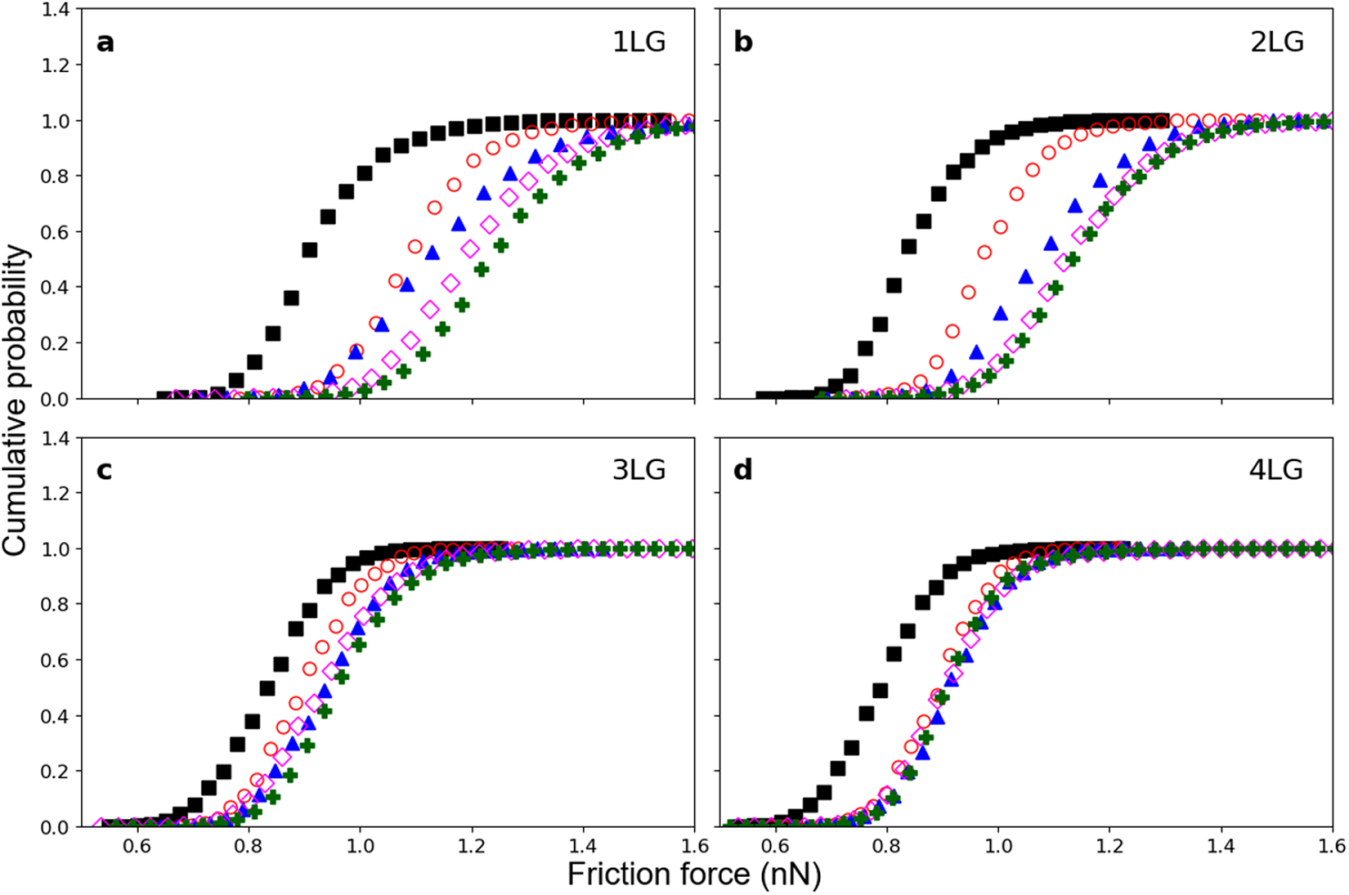
Cumulative distribution of friction forces for (**a**) 1LG, (**b**) 2LG, (**c**) 3LG and (**d**) 4LG at different scanning velocities. Scanning velocities are: 1 µm/s (black squares), 5 µm/s (red circles), 12 µm/s (blue triangles), 22 µm/s (magenta diamonds) and 30 µm/s (green crosses).

In Fig. 3, the cumulative probability of friction forces at different scanning velocities are presented for the monolayer and multilayer samples. The cumulative probability curves show the probability of a slip event occurs at a given friction force. At low scanning velocities, 5 μm/s and below, the onset of slip events is observed at about the same force (∼0.9 nN) for the monolayer (Fig. 3a) and multilayer samples (Fig. 3b–d) while the maximum slip probability is achieved at much higher forces for the monolayer (∼1.4 nN) than the multilayer graphene (∼1.0 nN). As velocity is increased, for a monolayer there is a distinction in the probability curves, while for multilayer the curves overlap tending to one single curve. At high scanning velocities, above 22 μm/s, the onset of slip is observed at a much higher forces for the monolayer (∼1.0 nN) and bilayer (∼0.9 nN) than for the additional three- and four-layer graphene (∼0.7 nN) while the maximum slip probability is observed to decrease with the number of layers being ∼1.6 nN for the monolayer, ∼1.4 nN for the bilayer, ∼1.2 nN for the three-layer and ∼1.1 nN for the four-layer sample.

**Figure 4:**
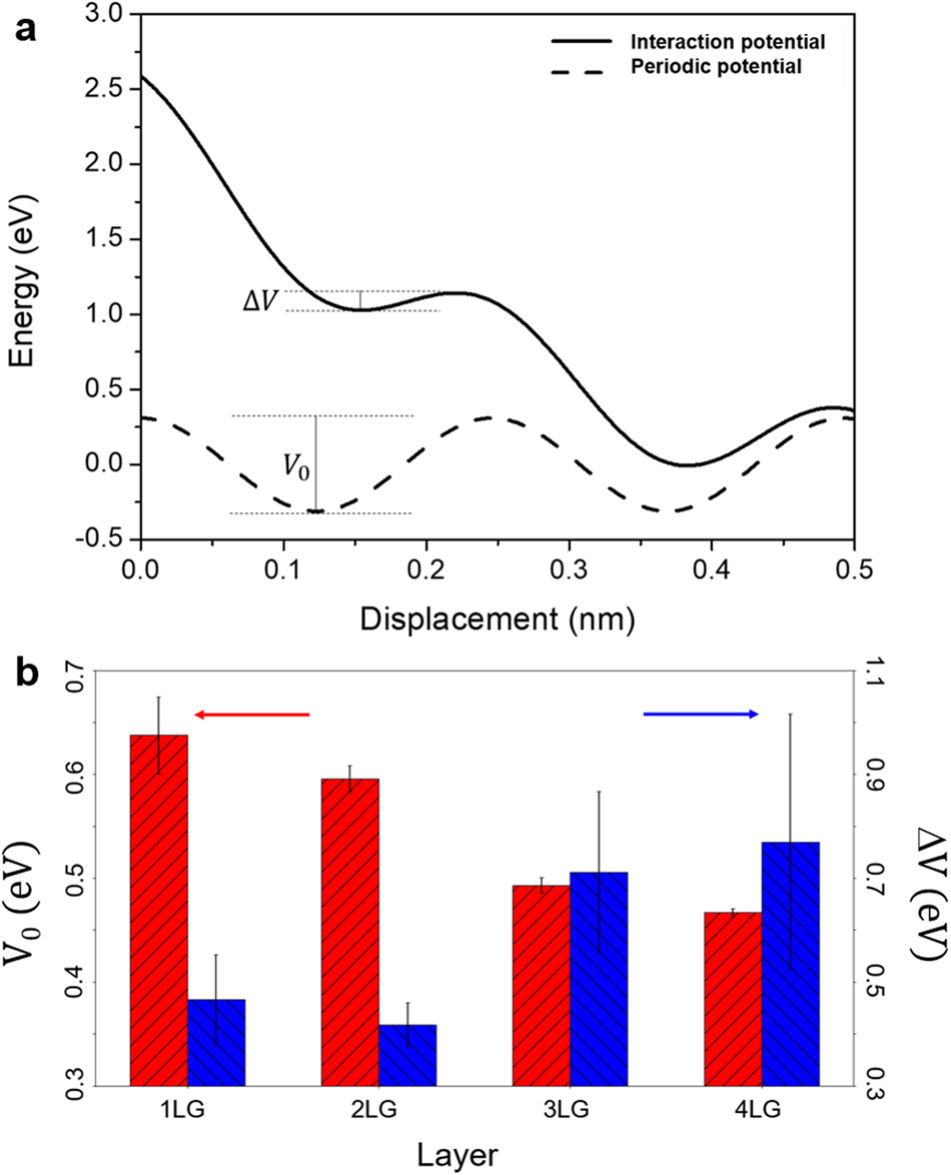
(**a**) Tip-sample interaction potential (solid line) and periodic potential (dashed line) due to the sample surface. (**b**) Amplitude of the periodic potential *V*_0_ (red bars) on the left, calculated with the estimated critical force *F*_*c*_ and potential barrier *ΔV* (blue bars) for the four different layers on the right. Error bars are the confidence interval of the fitting result.

In Fig. 4a, we show the total interaction potential *V(x*, *t)* along with the energy barrier Δ*V*; and the sinusoidal potential due to the periodicity of the sample with amplitude *V*_0_. In Fig. 4b the estimated amplitude of the *V*_0_ and the Δ*V* are shown. While the amplitude diminishes with the increasing number of layers from 0.7 to 0.5 eV the barrier height increases from 0.5 to 0.8 eV.

### Contact resonances between tip and graphene

**Figure 5:**
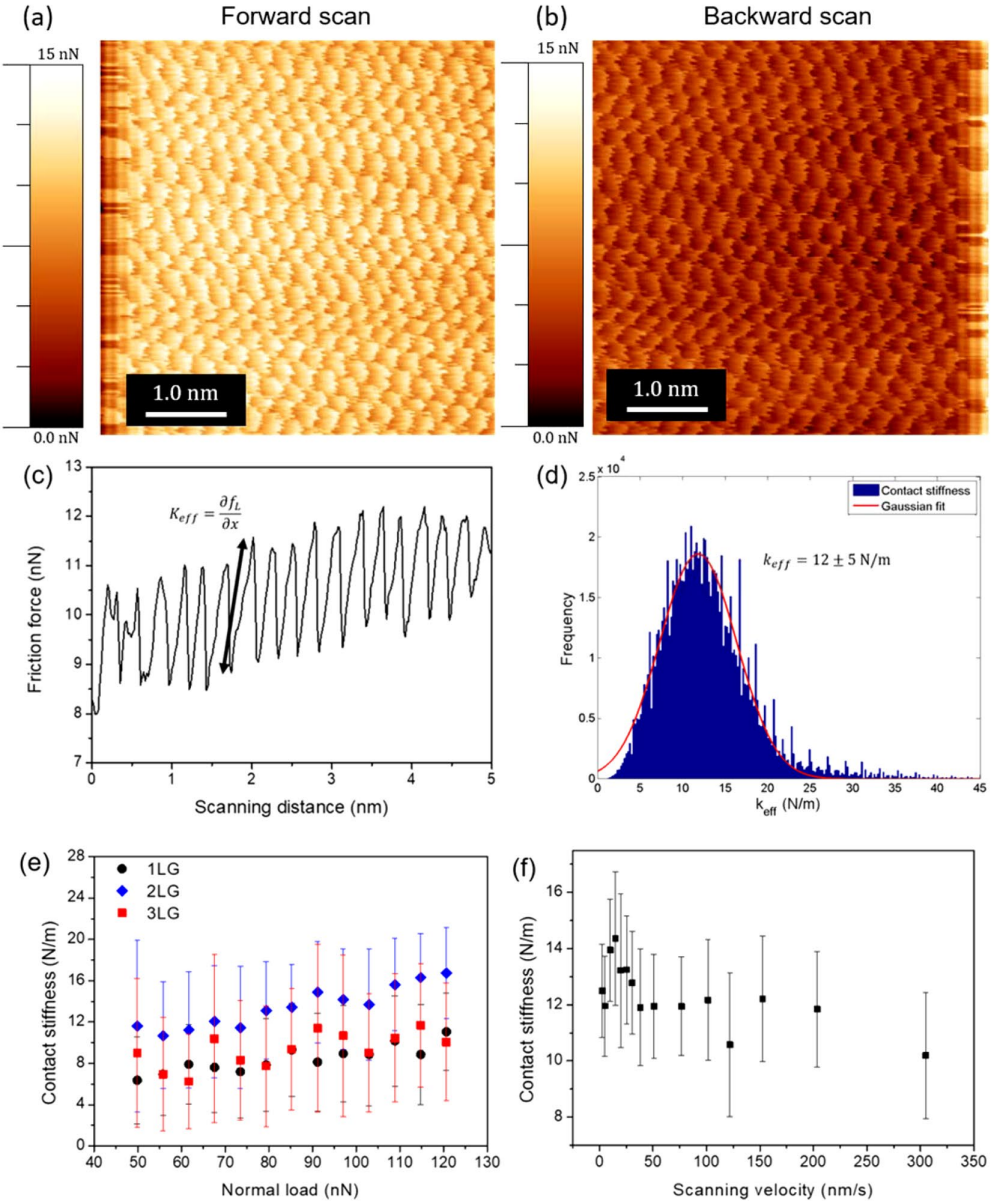
Lattice resolution images used to calculate the effective contact stiffness *k*_*eff*_. (**a**) Forward scan; (**b**) Backward scan. (**c**) A stick-slip profile. The slope of the stick part is the effective stiffness. (**d**) Histogram of the calculated *k*_*eff*_. (**e**) Measured contact stiffness, for three different graphene layers, as a function of normal load. (**f**) Stiffness measured for the different scanning velocities in a monolayer. The contact stiffness *k*_*eff*_ is also independent of the scanning velocity.

The effective contact stiffness *k*_*eff*_ between sample and cantilever tip was calculated from stick-slip profiles, measured in friction images with lattice resolution, to be 12 ± 5 N/m as shown in Fig. 5a–d. No significant differences in *k*_*eff*_ with the increasing normal load and number of layers (Fig. 5e) or scanning velocities (Fig. 5f) was observed.

**Figure 6:**
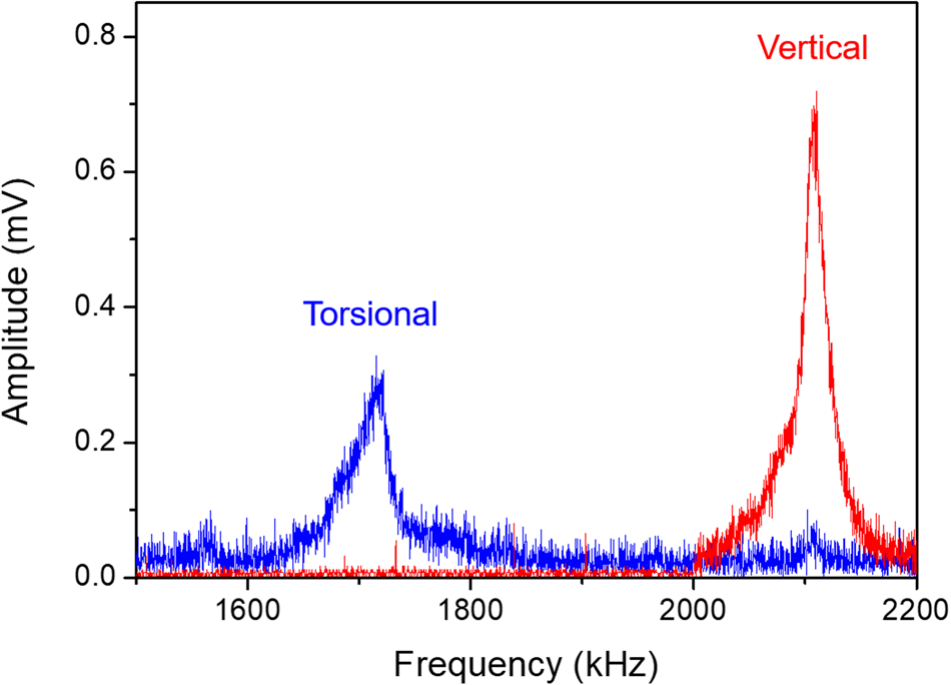
Torsional resonance spectrum of the cantilever-graphene system. In blue, a torsional resonant peak is observed at ~1.7 MHz, while in red, the vertical resonant peak is observed at ∼2.1 MHz.

The attempt frequency of a slip event, *f*_0_, remains roughly the same (∼1.8 MHz) for the four different graphene layers, as seen in Table 1. To check if such frequency is associated with any resonance of the cantilever tip in contact with the sample, we measured both the vertical and torsional resonance spectra of the cantilever-sample system. In Fig. 6 we are presenting the spectra in the range of 1.5 to 2.2 MHz while the full spectra can be seen in the supplemental material. A torsional resonance peak of the system was measured at ∼1.7 MHz with a full width at half-maximum (FWHM) of ∼40 kHz while a peak associated with the vertical resonance of the system was observed at 2.1 MHz with a FWHM of ∼30 kHz.

## Discussion

The decreasing in friction with the increasing number of layers, as seen in Fig. 1, is in good agreement with results reported on graphene tribology literature^12,15,28^. The dependence of friction with the number of layers as observed in experiments involving the sliding of an AFM tip over the graphene surface is an intricate problem. In such a system, with a significant number of atoms at the contact interface, the distinction in friction at different layers has shown to have contributions from the electron-phonon interaction^12^, the out of plane deformation of graphene at the tip contact, known as puckering^15^, and the surface corrugation at the interface^28^. Our experiments, measuring friction as a function of velocity for the monolayer and multilayer graphene, do not exclude the contribution of any of these processes, but show that the conversion of kinetic energy of the tip into electrons and phonons excitation, or even corrugation of the surface at the contact, is influenced by velocity.

In a solid-to-solid contact, the relationship between friction and velocity is influenced by temperature and mechanical resonances of the systems in contact. They affect the probability of the atoms, at the mechanical contact, to jump between minima of the interaction potential. That relationship is not fully understood and, as shown in Fig. 1, their study in monolayer or bilayer materials seems to be interesting, as the influence of velocity in friction is more pronounced than the influence observed at three or more layers.

Early studies on bulk crystal surfaces suggest a linear dependence of the friction force with the natural logarithm of the scanning velocity^31^. Later, it has been shown that this linear increase occurs until a critical saturation point where friction becomes constant^33,35^. In our case, Fig. 2 shows that for both mono and bilayer graphene, there is a pronounced linear increase of friction with the logarithm of the scanning velocity and the friction force reaches a critical point at high velocities, while for three- and four-layer graphene, the saturation occurs at lower velocities. Friction saturation occurs when thermal energy is no longer assisting the tip to overcome the potential barrier between two potential minima. We show that for monolayer and bilayer thermal effects are still very much active even at high scanning speeds. Also, the friction-velocity curves have a higher slope than the curves for three- and four-layers, indicating that a more corrugated potential is involved in the friction process^33^.

One must note that our experiment differs considerably from previous reports in literature by two conditions: I) we are dealing with atomically thin materials, while other studies were performed on bulk NaCl^31^, mica^33^ and graphite^36^; II) our experiments were performed in ambient air. The significance of the first condition resides in the fact that atomically thin materials are more easily deformed than bulk materials. Once the graphene sheet is deformed, the contact area increases as the tip scans the surface, therefore increasing the friction force and work done by the tip to overcome the energy barrier. For three- and four-layers, this deformation is significantly lower than for monolayer and bilayer, and less work is necessary for the tip to overcome the potential barrier. Consequently, the velocity influence in friction increases with the decrease in layers. Moreover, the energy dissipated during scanning may induce additional out of plane corrugations in the mono- and bilayer graphene. It has been shown that temperature indeed plays a significant role in the tribological behavior of graphene, as the temperature increases so does the surface roughness and the graphene sheet deformation^42^. As for the second condition, a consequence could be a correlation between adhesion and the measured friction, however previous results do not reinforce this idea^43,44^.

Computer simulations have also been used to study the influence of the scanning speed in friction at graphene sheets^24,26^. In contrary to our results, Smolyanitsky and coworkers^24^ observed a non-linear behavior for monolayer, while Li and coworkers^26^ observed no influence of the sliding speeds in the stick-slip friction profiles. However, such simulations were performed at scanning velocities in the range of a few m/s, while our experiments are limited to speeds of the order of µm/s.

The cumulative probabilities shown in Fig. 3 supports the large difference in friction between monolayer and multilayer graphene with velocity. At low scanning velocities the lateral forces necessary to initiate slip are about the same and independent from the number of layers. The influence of the number of layers in the friction forces at the onset of slip is significantly enhanced at high scanning velocities. That trend is observed as well for the average friction forces shown in Fig. 2, as the difference in friction between the different number of layers is much smaller at low than at high scanning velocities. At the low scanning velocities, we attribute the difference in friction between the monolayer and multilayer graphene samples to puckering^15^. As velocity is increased, the additional contribution to friction, leading to a clear distinction in data for the monolayer and multilayer, may be attributed to the influence of energy dissipation during the sliding of the tip that possibly introduces additional roughening of the graphene^42^.

We calculated both amplitude of the sinusoidal potential as well as the potential barrier for the graphene layers, as shown in Fig. 4b. The potential amplitude is directly related to the critical force^33,45^, thus also decreasing with the increasing number of layers. The barrier, however, considers the lateral force exerted by the cantilever as it is deformed by the scanning tip. Friction forces are larger for the mono- and bilayer, increasing the tip torsion and the elastic energy stored in the cantilever. That diminishes the potential barrier. Previous measurements of the barrier height done for graphite using stick-slip statistics^46^ and temperature variation^47^ shows that the barrier should be around 0.1 and 0.2 eV, which is lower than obtained in our experiments. One significant difference in our work is related to the normal contact load, as we used a much higher load, ∼110 nN, than the previously mentioned experiments, and the energy barrier is sensitive to changes in the applied load^33^.

The contact stiffness is indistinguishable between the measured graphene layers. As the contact stiffness is related to elastic properties of the materials^48^, our results suggest that graphene in-plane elasticity is similar for different number of layers. This agrees with previous studies on graphene that have measured elastic properties in sheets up to three layers and showed that although bilayer graphene needs higher loads for the same amount of deflection as a monolayer, the elastic moduli for all three layers are the same within the experimental error^13^.

With the fitted parameters, we calculated the attempt frequency of a slip event, which might be associated with a torsional resonance of frequency of the cantilever^33^. Our spectrum shows a torsional resonance peak close to the calculated parameter for monolayer, suggesting that the attempt frequency is possibly driven by the torsional resonances of the cantilever tip in contact with graphene. Thus, thermal energy may also contribute to the friction-velocity relation by reducing friction while driving the cantilever tip and graphene system to oscillate at resonance. Additional vibrations lead to the anticipation of the slip of the tip across the surface facilitating the movement of the nanoscale asperities in contact^49^. Thus, by controlling important parameters on the resonance of the cantilever, one could facilitate the friction process.

## Conclusions

We studied friction mechanisms on monolayer and multilayer graphene with an AFM, focusing on probing the friction-velocity relation for different number of graphene layers. Friction in graphene shows to be layer dependent. Friction at the monolayer was significantly influenced by the sliding velocity and a constant critical friction force was obtained only at high scanning speeds, evidencing the influence of temperature in the process. Possibly, energy dissipated by friction may induce additional out of plane corrugation increasing the friction forces. The influence of velocity in multilayer graphene is much less pronounced and critical friction forces were observed at much lower values. Our results are in good agreement with the thermally activated Prandtl-Tomlinson model predictions. We were able to interpret our data with the PT model and extract important parameters in the friction mechanisms, such as the interaction potential barrier, the critical force at which the barrier vanishes, and the attempt frequency of the slip events. The amplitude of the potential due to the periodicity of graphene decreases as the number of layers increases and with it the critical force. The potential barrier, however, increases with the number of layers, due to the force exerted by the cantilever. The attempt frequency was associated with the torsional resonance of the cantilever in contact with the graphene. Consequently, thermal vibrations of the mechanical system facilitate the sliding of the tip reducing friction during movement of the nanoscale tip in contact.

## Methods

### Sample preparation

Graphene samples were obtained by exfoliation of highly oriented pyrolytic graphite (HOPG) crystals with the use of a scotch tape. Graphite flakes adhered to the tape were mechanically transferred to a Si substrate with a 300 nm oxide layer on top. The samples were observed by optical microscopy and the number of graphene layers at the flakes were determined by Raman spectroscopy (Alpha 300, WITec Wissenschaftliche Instrumente und Technologie GmbH, Germany). For details in identification of the number of layers, see supplementary information.

### Friction force measurements

The topography images and friction force as a function of scanning velocity were measured from images obtained with the Nanowizard AFM (JPK Instruments A. G., Germany). The effective contact stiffness *k*_*eff*_ between sample and cantilever tip were measured from lattice resolution images of 5 nm × 5 nm obtained with the MultiMode Nanoscope IIIa AFM (Brukers, USA). Silicon nitride V-shaped cantilevers, with tip radius of ∼10 nm, normal and torsional spring constant of 0.40 ± 0.01 and 86 ± 4 N/m respectively were used. The AFMs were calibrated and the friction forces were obtained from the product between the cantilever’s torsional spring constant and the lateral displacement of the tip measured in the AFM photodetector^50^. The measurements were taken at a normal applied load of ∼110 nN. The scanning velocities ranged from 0.4 to 50.0 µm/s. The scanning direction was kept perpendicular to the cantilever’s main axis. All measurements were performed at ∼25 °C and a relative humidity of ∼60%. Even after scanning the same areas several times, no wear was observed. To interpret our results on friction as a function of scanning velocity with the PT model, we have fitted our data with Eq. (4) using a Levemberg-Marquardt algorithm^51^ considering *ΔV/kT*, *F*_*c*_ and *ln v*_0_ as free parameters.

### Contact resonance measurements

The normal and torsional contact resonances of the cantilever-graphene system were acquired with the use of a piezoelectric ceramic actuator, a function generator (AFG 3021, Tektronix, Inc., U.S.A.), and a lock-in amplifier (SR844, Stanford Research Systems, U. S. A.). The sample was positioned on top of the piezoelectric actuator and, at a constant drive amplitude, the frequency of the actuator was varied between 25 kHz to 5 MHz while normal and torsional amplitude signals of the cantilever in contact with the surface were measured by the lock-in.

## Supplementary information

Supplementary information file

## Data Availability

The datasets generated and analyzed during the current study are available from the corresponding author on reasonable request.
