# Supplementary material for: Velocity-dependent friction enhances tribomechanical differences between monolayer and multilayer graphene
Source: Sci Rep. 2019 Oct 10;9:14555. doi: 10.1038/s41598-019-51103-1 (PMC6787015; doi:10.1038/s41598-019-51103-1)
Supplement: Supplementary file 1 — Supplementary information file [file 41598_2019_51103_MOESM1_ESM.pdf]

Supplementary information

Velocity-dependent friction enhances tribomechanical differences between monolayer and multilayer graphene

F. Ptak[1], C. M. Almeida[2] & R. Prioli[1]

[1] *Departamento de Física, Pontifícia Universidade Católica do Rio de Janeiro, Marques de São Vicente 225, Rio de Janeiro, 22453-900, Brazil*

[2] *Divisão de Metrologia de Materiais, Instituto de Metrologia, Normalização e Qualidade Industrial (INMETRO), Av. Nossa Senhora das Graças 50, Xerém, Duque de Caxias, Rio de Janeiro, 25250-020, Brazil*

Identifying the number of graphene layers

To identify the number of graphene layers, Raman spectroscopy was performed. Two main peaks are identified in Raman spectra at ~ 1580 cm^{-1} and ~ 2700 cm^{-1}, G and 2D bands, respectively. The shape of 2D bands is used to identify the number of layers in graphitic materials, from one to up to 5 layers graphene[S1]. In graphene monolayer, the 2D band is a single and sharp peak, while in bilayer it consists of four overlapped peaks. The shape of the 2D band evolves, and for more than 5 layers, it exhibits 2 overlapped peaks with roughly 1/4 and 1/2 of the maximum height of the G peak. Figure S1 shows the Raman spectra for the monolayer and multilayer graphene, used to confirm the number of sheets on flakes used in our experiments.

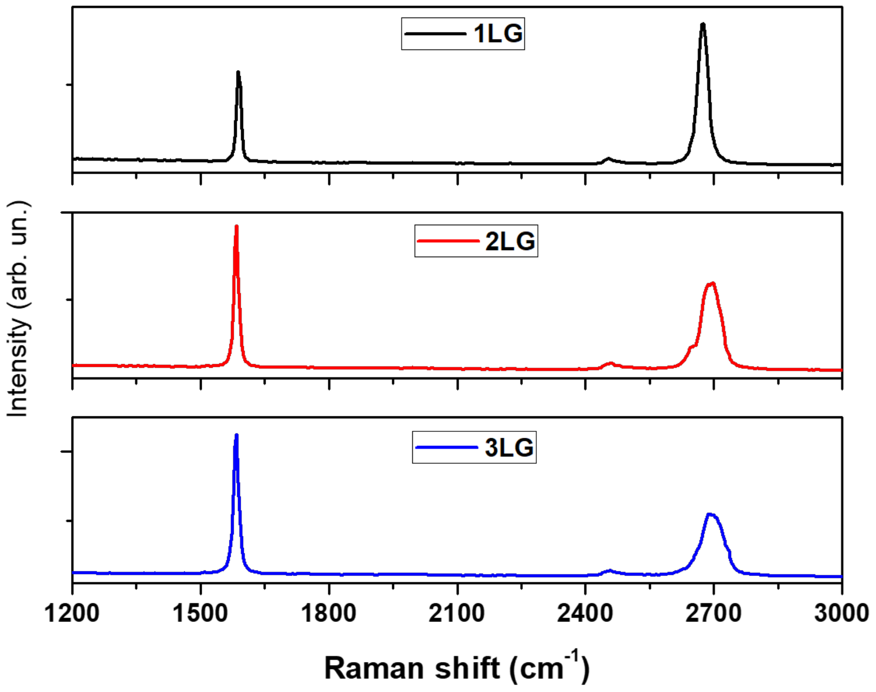

Figure S1: Raman spectra of the graphene sample. At the top the spectrum of a monolayer (1LG). The middle spectrum is for a bilayer (2LG) while the bottom spectrum is for a three-layer graphene (3LG).

The number of graphene layers in the flakes studied in this work were confirmed by AFM. Height histograms performed on topography images, as show in figure S2 were used to confirm the number of layers and measure the step height between them. The step heights observed as 1.34 ± 0.12 nm between the substrate and monolayer, 0.59 ± 0.12 nm between 1LG and 2LG, 0.53 ± 0.09 nm between 2LG and 3LG, and 0.52 ±0.10 nm for 3LG to 4LG in agreement with the literature[S2, S3].

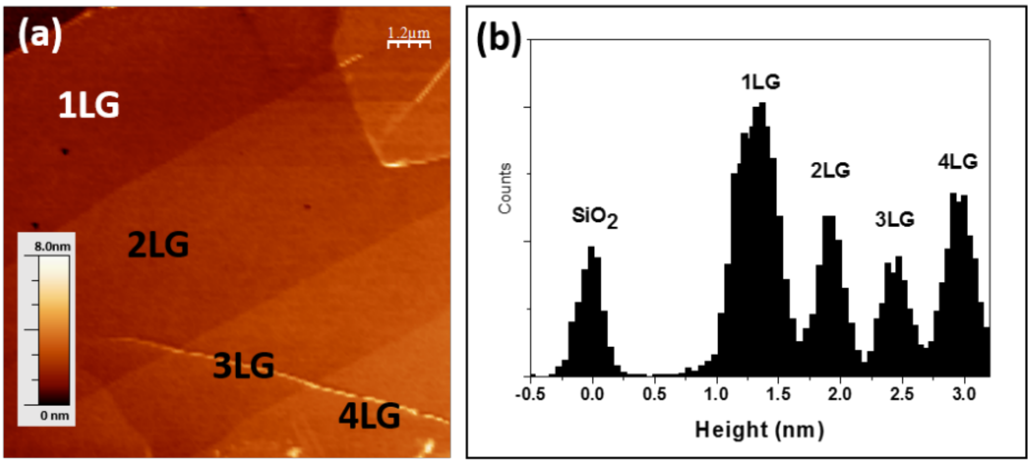

Figure S2: (a) AFM topography image of an analyzed 1LG, 2LG, 3LG and 4LG region with (b) the histogram of relevant regions in (a).

Energy dissipated during the friction process

The dissipated energy was calculated following the method of Reference S4. With the increasing scanning velocity, we calculated energies up to ~ 2.0 eV for 1LG and ~ 1.5 eV for 4LG. It is directly proportional to the mean measured friction force, thus it is possible to see a saturation of energy with a certain scanning velocity.

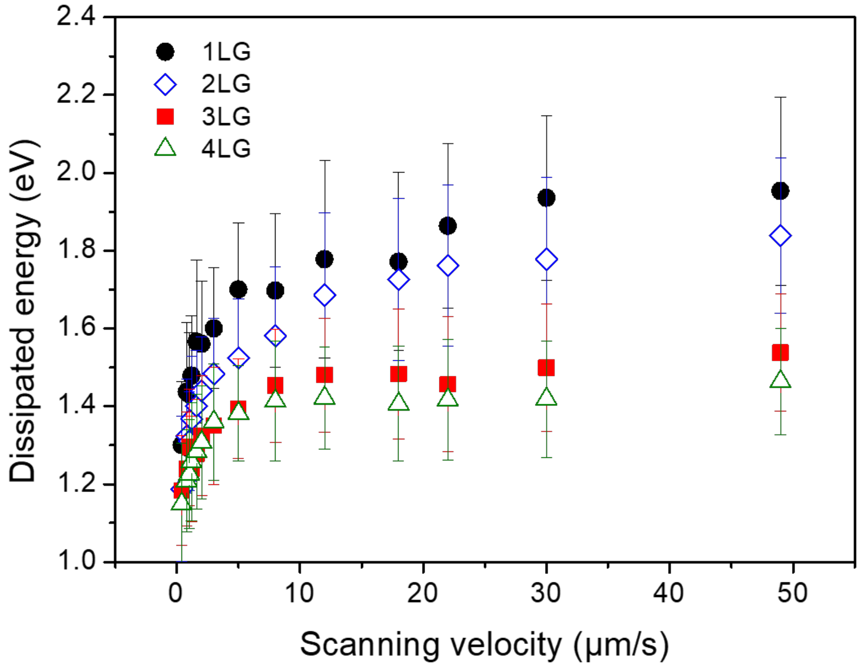

Figure S3: Calculated dissipated energy with increasing scanning velocity.

As the lateral force constant of the used cantilever is greater than the effective contact stiffness k_{eff}, the energy is released both on the cantilever and irradiated as elastic waves on the graphene's crystal lattice, exciting the carbon atoms, therefore increasing the temperature on the process. It is worth noting that the thermal conductivity is much higher for graphene than for silicon (the cantilever) and silicon nitride (the tip) [S5], causing most of the energy to be dissipated on the graphene lattice.

Graphene/cantilever contact resonance

Figure S4 shows both the torsional (a) and vertical (b) resonance spectrum of the cantilever in contact with a monolayer graphene, using the method described in Reference S6. Both spectrums were acquired using the same drive amplitude.

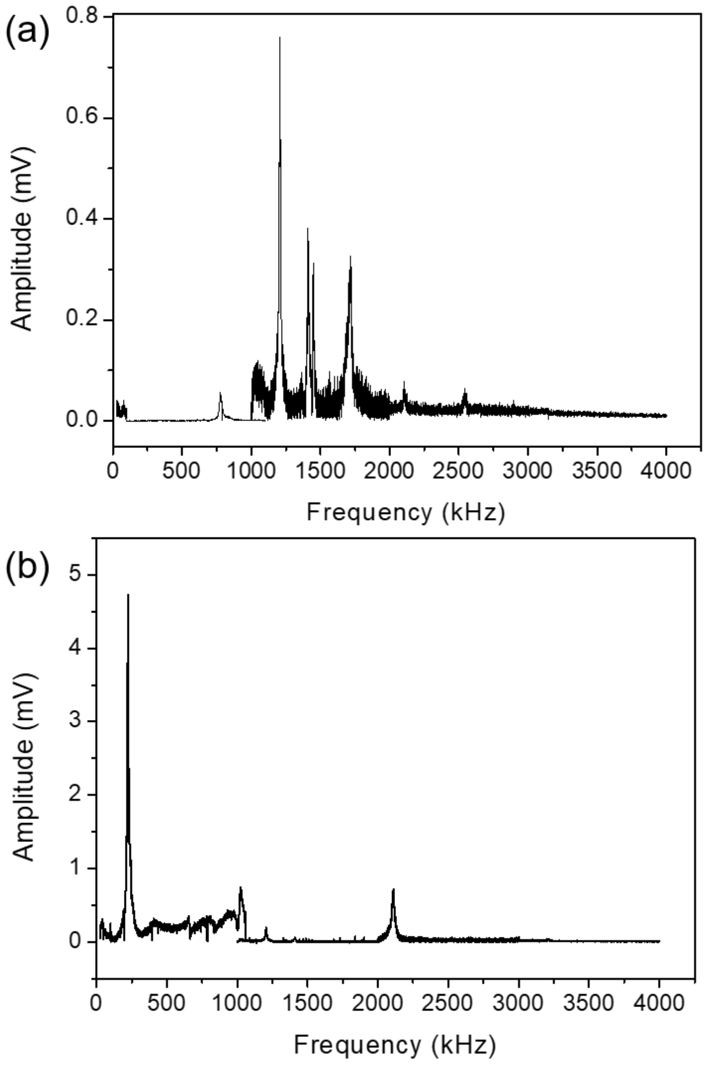

Figure S4: Full resonance spectrum of the cantilever/graphene contact system. (a) Torsional deflection spectrum; (b) Vertical deflection spectrum.

The torsional spectra show the first three antisymmetrical modes of resonance from the triangular cantilever while the vertical spectra show the first two symmetrical resonances that leads to bending of the triangular cantilever. Torsional peaks can be found at f_1 = 0.8 MHz, f_2 = 1.2 MHz, f_3 = 1.4 MHz, f_4 = 1.7 MHz, f_5 = 2.1 MHz and f_6 = 2.5 MHz. Normal bending peaks are f_1 = 37.8 kHz, f_2 = 223.7 kHz, f_3 = 1.2 MHz, f_4 = 1.4 MHz, f_5 = 2.1 MHz and f_6 = 3.2 MHz.

References

S1. Ferrari, A. C. *et al.*, Raman spectrum of graphene and graphene layers. *Phys. Rev. Lett.* **97**, 187401 (2006).

S2. Nemes-Incze P., Osváth Z., Kamarás K., & Biro L. P. Anomalies in thickness measurements of graphene and few layer graphite crystals by tapping mode atomic force microscopy. *Carbon* **46**, 1435-1442 (2008).

S3. Shearer, C. J., Slattery, A. D., Stapleton, A. J., Shapter, J. G., & Gibson, C. T. Accurate thickness measurement of graphene. *Nanotechnology* **27**, 125704 (2016).

S4. Colchero, J., Baró, A. M. & Marti, O. Energy dissipation in scanning force microscopy - friction on an atomic scale. *Tribol. Lett.* **2**, 327-343 (1996).

S5. Balandin, A. A. *et al.* Superior thermal conductivity of single-layer graphene. *Nano Lett.* **8**, 902 (2008).

S6. Killgore, J. P. & DelRio, F. W. Contact resonance force microscopy for viscoelastic property measurements: from fundamentals to state-of-the-art applications. *Macromolecules* **51**, 6977 (2018).
